# Patient-reported outcomes in major depressive disorder with suicidal ideation: a real-world data analysis using PatientsLikeMe platform

**DOI:** 10.1186/s12888-020-02758-y

**Published:** 2020-07-23

**Authors:** Stephane Borentain, Abigail I. Nash, Rachna Dayal, Allitia DiBernardo

**Affiliations:** 1 Janssen Research & Development, LLC, Titusville, NJ USA; 2 Janssen Scientific Affairs, LLC, Titusville, NJ USA

**Keywords:** PatientsLikeMe, Major depressive disorder, Major depressive disorder with suicidal ideation, Suicidal ideation

## Abstract

**Background:**

The current analysis utilized data collected via an online patient community platform, PatientsLikeMe (PLM) to compare patient-reported experiences in patients with major depressive disorder (MDD) with suicidal ideation (MDSI) to those with MDD but without suicidal ideation.

**Methods:**

PLM members who joined PLM between May-2007 and February-2018 and reported a diagnosis of MDD were included. The MDSI cohort included patients with MDD who reported at least one suicide-related symptom at a severity greater than “none”. Demographics, comorbidities, symptoms, and side-effects were compared between MDSI and MDD cohorts. Factors correlated with suicidal ideation (SI) were determined by a random forest procedure.

**Results:**

Patients in the MDSI cohort (*n* = 266) were younger (median age, 36 vs 44 years) with an earlier disease onset (before 30 years, 83% vs 71%), and a longer diagnosis latency (median, 4 vs 2 years) vs patients in the MDD cohort (*n* = 11,963). Majority of patients were women in both cohorts (73% vs 83%). Median number of psychiatric comorbidities was higher in the MDSI cohort (4 vs 3). Unprompted symptoms (e.g., loneliness, feeling of hopelessness, social anxiety, impulsivity, and self-hating thoughts) were more frequent in the MDSI cohort. Hopelessness, loneliness, anhedonia, social anxiety, and younger age were highly correlated with suicidal ideation.

**Conclusions:**

This analysis utilized patient-reported data to better understand symptoms, experiences, and characteristics of patients with MDSI compared to patients with MDD. The results identified various risk factors correlated with suicidal ideation that may help guide clinical judgement for patients with MDD who may not voluntarily report suicidal ideation.

## Background

Suicide is a major public health concern and is one of the leading causes of death worldwide [1]. In the U.S., 47,000 deaths were reported in 2017 due to suicide. Of the 17.6 million patients with major depressive disorder (MDD), 5.5 million adults reported suicide ideation (SI) and 2 million patients reported SI with intent [2]. In the same year, age adjusted suicide rate was 15.3 per 100,000 persons, considerably higher than the global rate of 10.5 per 100,000 persons [3, 4]. Notably, the highest prevalence of suicide is found in the most economically productive age group (15–44 years) [5]. Moreover, examination of data of young adults aged 18–25 years from the National Surveys on Drug Use and Health (2009–2015) showed an increase in the 12-month prevalence of SI (6.1 to 8.3%), suicide plan (2.0 to 2.7%), and suicide attempt (1.1 to 1.6%) [6].

Suicidal behavior and ideation have a significant economic burden. In Europe, Jaffe et al. [7] reported SI to be associated with higher absenteeism, presenteeism, work productivity impairment, and activity impairment compared to individuals without SI. The burden of suicidal behavior and ideation extends to caregivers of individuals with SI, as they reported that the constant worry affected their social and occupational functioning [8]. In addition to the traumatic, often unexpected loss of life, friends and family members of an individual who dies by suicide may face social isolation, stigma, and are at increased risk for depression-related mental illness, substance abuse, and suicide [9].

MDD is the mental health diagnosis most commonly associated with suicide. Globally, more than 60% of individuals who have attempted suicide struggle with MDD [10]. Moreover, patients with MDD have a 20-fold higher risk of suicide than the general population [11]. As the prevalence of SI is high among patients with MDD, understanding the characteristics that differ in patients with MDD and SI compared to those without SI is critical for early identification of high-risk patients [12].

Detection of SI, early diagnosis of depression, continuity of care and treatment adherence have been identified as critical to improve outcomes in patient with SI [13]. Therefore, it is important to understand the patient perspective and the needs of this high-risk population to increase efficacy of suicide prevention strategies. Comorbid psychiatric conditions such as personality disorders, anxiety disorders, and substance use disorders are associated with an increased risk of suicide in patients with MDD [14–17]. Hence, the treatment plan should also feature concomitant treatment of the comorbid condition. In patients with comorbid substance use disorders, easing access to treatment, providing counseling, and social support in addition to the treatment of depression is recommended [15]. However, as patients with SI are often excluded from most antidepressant trials, limited data are available to study their disease or treatment characteristics [18].

Online patient communities provide patients with a platform to share their disease and treatment experiences, which subsequently provide researchers with valuable data pertaining to patient reported outcomes, perspectives, and preferences [19]. PatientsLikeMe (PLM) is one such global online community that provides patients with resources to record, track, and share their symptoms, disease experiences, and treatment outcomes, and to improve their care through peer-to-peer interactions. Patients are free to share as much or as little data as they want. PLM enables visualization of an individual’s health profile graphically as well as aggregates data into reports that are available on the website [20]. The PLM community comprises > 775,000 patients overall from across the globe, with > 62,100 members reporting a diagnosis of MDD (as of 23rd May 2020) [21]. In the present analysis, we utilized the data provided by PLM to characterize and compare the patient-reported characteristics and experiences of living with MDD with SI (MDSI group) to those with MDD but without SI (MDD group).

## Methods

### Study population

Retrospective data of adult patients (aged ≥18 years) who were members of the global PLM community, registered to the PLM website between May 2007 and February 2018, and reported a diagnosis of MDD were obtained. Although patients in the PLM community were not prompted to report any symptom related to SI, those patients who reported at least one SI-related symptom (“suicidal thoughts or urges,” “suicidal,” “depression with suicidal thoughts,” “SI,” “suicide attempt,” or “suicidal behavior”) at a severity greater than none were included in the MDSI cohort. Patients who reported an MDD diagnosis without any suicide-related symptoms were included in the MDD cohort. Patients who reported a primary diagnosis of bipolar disorder, borderline personality disorder, paranoid schizophrenia, Parkinson’s disease, post-traumatic stress disorder, psychosis disorder, psychotic depression, schizoaffective disorder, schizoid personality disorder, schizophrenia, or any life-threatening illness (e,g., cancer) in the PLM database were excluded (Supplementary Table 1). Patients with active or prior history of substance abuse/dependence were not excluded.

### Assessments

All assessments were performed based on the clinical, diagnostic, and treatment experiences reported by patients with MDD (with and without SI) on the PLM platform. Direct, spontaneous data reported by the patients were used and no validated instruments were employed for data collection. The data collected from patients were categorized into demographics, comorbid conditions, symptoms, and treatments. Demographic data of patients included age, sex, race/ethnicity, nationality, education level, and health insurance type, while comorbidities and condition-associated information included diagnosis status, onset date (date of first symptom), and diagnosis date. The prevalence of each comorbidity was also assessed.

Symptoms reported by the patients on the PLM platform were categorized into three groups: prompted general symptoms (pain, fatigue, insomnia, depressed mood, and anxious mood), MDD-specific symptoms (increased need for sleep, anhedonia, decreased/increased appetite, irritability, bradykinesia, and decreased sex drive), and spontaneously-reported symptoms (any symptom the patient wishes to report and track on their profile). Recording the severity of the five general symptoms was mandatory for patients with MDD on the PLM platform and severity reporting was also mandated for any MDD-specific or spontaneously reported symptoms the patient chose to include. Symptom severity was recorded on a subjective rating scale, by the patient, as none, mild, moderate, and severe.

All patients on the PLM platform who reported an MDD diagnosis were prompted to report whether they have ever been prescribed any of the following MDD-associated treatments: bupropion, citalopram, duloxetine, escitalopram, fluoxetine, sertraline, venlafaxine or individual therapy. Additionally, patients also provided treatment related details including treatment dose, start date, perceived effectiveness, side effects and their severity, and burden of treatment. Perceived treatment effectiveness was recorded on a subjective rating scale, by the patient, as “can’t tell,” “none,” “slight,” “moderate,” and “major,” while severity of side effects was rated as “none,” “mild,” “moderate,” and “severe.”

Patients were also encouraged to complete a mood map platform survey (PLM’s proprietary 25-item survey, which captured information that might reflect the patient’s current mood) as well as freely enter text in forums, treatment evaluation reports, and treatment stop reports.

### Risk factors for suicidal ideation

We used a random forest classifier (RF) to determine the risk factors for SI in the study population. A RF is a data classification algorithm comprising multiple individual decision trees that operate as a group or ensemble. Each tree gives a class or variable prediction and the one with the most votes becomes the model’s prediction [22]. RF has advantages over other data classification methodologies such as the ability to handle highly non-linearly correlated data, robustness to noise, tuning simplicity, and opportunity for efficient parallel processing [23]. We developed and trained the RF model to predict whether a patient belongs to the MDSI cohort or MDD cohort. Demographic, comorbidity, symptom, and mood map variables were assessed to determine the most important characteristics in distinguishing the cohorts.

### Statistical analysis

Descriptive statistics were used to summarize the data. Categorical variables were presented as frequency and numbers while continuous variables were presented as medians.

A chi-square test of independence was applied to compare sets of distributions, whereas a binomial test of proportions was used for pairwise comparison of binary proportions. Bonferroni correction was applied to control for potential Type I errors related to multiple comparisons. Variability around proportions was presented by 95% confidence intervals (CI). CIs of binary proportions were calculated using Clopper-Pearson method, which yields asymmetric upper and lower bounds that do not exceed 0 or 1.

## Results

### Patient characteristics

Of the 12,229 PLM users with MDD who registered and provided data during the study period, 266 reported symptoms related to suicidality with a severity greater than none (MDSI cohort), and 11,963 reported never experiencing any of these symptoms (MDD cohort).

The majority of the patients in both cohorts were White (MDSI: 87.5% vs. MDD: 86.7%, *p* = 0.3158) and had completed at least high school (MDSI: 91% vs. MDD: 94%, *p* = 0.3606). Patients in the MDSI cohort compared to those in the MDD cohort were significantly younger (median age 36 years vs. 44 years, *p* < 0.0001), more frequently men (27.1% vs. 16.7%, *p* < 0.0001), and were less likely to report a family history of MDD (45.9% vs. 49.7%, *p* = 0.0003) (Table 1).

**Table 1:** Baseline characteristics

Variable	MDSI (***n*** = 266)	MDD (***n*** = 11,963)
**Gender, n (%)**		
Total	258 (96.9)	11,553 (96.6)
Women	188 (72.9)	9622 (83.3)
Men	70 (27.1)	1931 (16.7)
**Age, years**		
Total	252 (94.7)	11,486 (96.0)
Median	36	44
**Race, n (%)**		
Total	136 (51.1)	8.970 (74.9)
White	119 (87.5)	7781 (86.7)
Mixed race	8 (5.9)	372 (4.1)
Black/African American	2 (1.5)	263 (2.9)
American Indian/Alaskan native	1 (0.7)	98 (1.1)
Asian	4 (2.9)	113 (1.25)
Native Hawaiian/other Pacific Islander	0 (0)	14 (0.2)
Preferred not to disclose	2 (1.47)	329 (3.7)
**Education, n (%)**		
Total	129 (48.5)	7510 (62.8)
8th grade or less (left school around 14 years of age)	0	35 (0.5)
Some high school, but did not graduate (left school around 16 years of age)	9 (7.0)	270 (3.6)
High school graduate or GED (left school around 18 years of age)	23 (17.8)	1161 (15.5)
Some college but less than a bachelor’s/undergraduate degree	51 (39.5)	3273 (43.6)
College bachelor’s/undergraduate degree	24 (18.6)	1650 (22.0)
Postgraduate degree (Master’s, doctorate, etc.)	19 (14.7)	952 (12.7)
Preferred not to answer	3 (2.3)	169 (2.3)
**Family history of MDD, n (%)**		
Total	218 (82.0)	6752 (56.4)
Yes	100 (45.9)	3353 (49.7)
No	92 (42.2)	2075 (30.7)
Don’t know	26 (11.9)	1324 (19.6)

**Abbreviations:***GED* General Education Development, *MDD* Major Depressive Disorder, *MDSI* Major Depressive Disorder with Suicidal Ideation

### Condition reports

#### Onset date and diagnosis latency

A total of 129 (48.5%) patients in the MDSI cohort and 6440 (53.8%) patients in the MDD cohort reported their first symptom date, while the diagnosis date was reported by 121 (46.0%) and 5943 (49.7%) patients in the MDSI and MDD cohorts, respectively. The MDSI cohort included a significantly larger proportion of users reporting an age of onset < 30 years (83.0% vs. 71.2%, *p* = 0.001) and significantly longer median diagnosis latency compared to the MDD cohort (4 years vs. 2 years, *p* = 0.0002).

#### Comorbidities

**Fig. 1:**
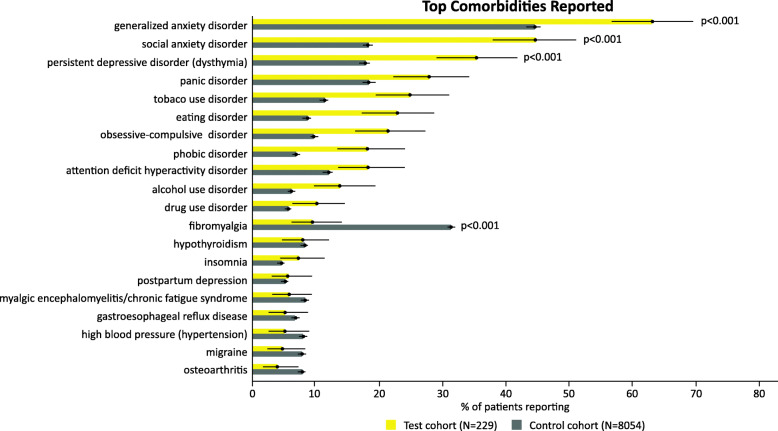
Top comorbidities

A total of 229 (86.1%) patients in the MDSI cohort and 8054 (67.3%) patients in the MDD cohort reported data on at least one comorbidity. The MDSI cohort reported a significantly greater median number of comorbidities than the MDD cohort (4 vs. 3, *p* < 0.0001). The top three comorbidities reported at a higher frequency in the MDSI cohort compared to the MDD cohort were generalized anxiety disorder (63.1%; 95% CI, 56.7–69.6% vs. 44.3%; 95% CI, 43.3–45.5%), social anxiety disorder (44.5%; 95% CI, 38.0–51.2% vs. 18.3%; 95% CI, 17.4–19.1%), and dysthymia (35.4%; 95% CI, 29.2–41.9% vs 17.7%; 95% CI, 16.9–18.6%) (*p* < 0.001 for all). However, the proportion of these comorbid conditions was relatively high in MDD cohort as well. Fibromyalgia was the only comorbidity that was reported significantly more in the MDD cohort than the MDSI cohort (31.0%; 95% CI, 30.0–32.0% vs. 9.6%; 95% CI, 6.1–14.2%, *p* < 0.001) (Fig. 1).

### Symptom reports

**Fig. 2:**
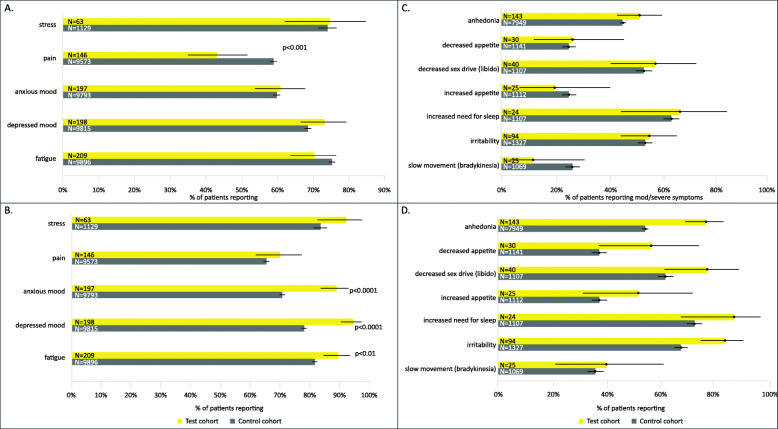
Median and worst scores of top symptoms reported. (**a**) Median scores of top prompted symptoms reported (**b**) Worst scores of top prompted symptoms reported (**c**) Median scores of MDD-specific symptoms (**d**) Worst scores of MDD-specific symptoms

All patients (MDSI: 266, MDD: 11,963) included in the study reported at least one symptom. Pain was the only prompted symptom that showed a significant difference in reporting frequency after averaging patient reports over time (MDSI: 43.2%; 95% CI, 35.0–51.6% vs. MDD: 58.9%; 95% CI, 57.9–59.9%, *p* < 0.001) (Fig. 2a). However, comparison of worst-ever scoring for prompted symptoms showed a significantly greater reporting frequency of “high severity” in the MDSI cohort than the MDD cohort for anxious mood (88.8%; 95% CI, 83.6–92.9% vs. 70.6%; 95% CI, 69.7–71.5%, *p* < 0.0001), depressed mood (94.4%; 95% CI, 90.3–97.2% vs. 78.0%; 95% CI, 77.1–78.8%, *p* < 0.0001), and fatigue (89.5; 95% CI, 84.5–93.3% vs. 81.6%; 95% CI, 80.8–82.3%, *p* < 0.01) (Fig. 2b).

No significant differences were observed between the two groups in the reporting frequency of MDD-specific symptoms after averaging patient reports over time (Fig. 2c). However, when the worst-ever scores were considered, significantly greater reporting frequency of “high severity” was observed in the MDSI cohort compared to the MDD cohort for anhedonia (76.9%; 95% CI, 69.2–83.6% vs. 54.6%; 95% CI, 53.5–55.7%, *p* < 0.0001) and irritability (84.0%; 95% CI, 75.0–90.8% vs. 67.8%; 95% CI, 65.2–70.3%, *p* < 0.001) (Fig. 2d). The prompted and MDD-specific symptoms’ results are supported by the mood map analysis. While symptom severities between the cohorts are similar when averaged over time (Supplementary Figure 1A), certain symptoms including depressed or anxious mood and irritability, do increase in severity when patients are at their worst (Supplementary Figure 1B).

**Fig. 3:**
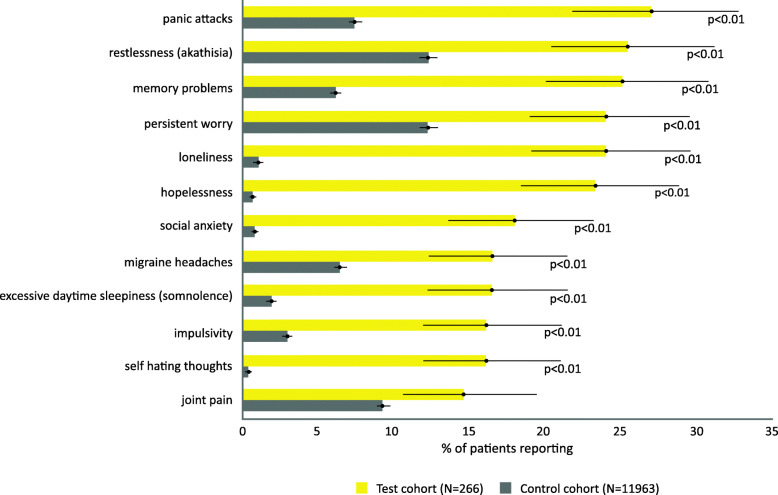
Top reported unprompted symptoms

Spontaneous symptom reporting frequency was significantly greater in the MDSI cohort compared to the MDD cohort for symptoms including panic attack (27.1%; 95% CI, 21.8–32.8% vs. 7.5%; 95% CI, 7.0–8.0%), restlessness (26.6%; 95% CI, 20.4–31.3% vs. 12.3%; 95% CI, 11.7–12.9%), memory problems (25.2%; 95% CI, 20.1–30.9% vs. 6.3%; 95% CI, 5.8–6.7%), loneliness (24.1%; 95% CI, 19.1–29.7% vs. 1.1%; 95% CI, 0.96–1.34%, *p* < 0.0001 for all), and persistent worry (24.1%; 95% CI, 19.1–29.7% vs. 12.3%; 95% CI, 11.8–12.9%, *p* < 0.001) (Fig. 3).

### Treatment reports

A total of 243 (91.4%) patients in the MDSI cohort and 10,951 (91.5%) patients in the MDD cohort reported receiving ≥1 treatment during the observational period. The MDSI cohort reported intake of more antidepressant treatments than the MDD cohort (mean values: 3 vs. 2.4). The five most reported treatments were sertraline (36.6%), bupropion (36.6%), fluoxetine (35.8%), venlafaxine (32.9%), and citalopram (31.3%) in the MDSI cohort, and bupropion (38.3%), fluoxetine (34.2%), sertraline (33.4%), duloxetine (32.0%), and venlafaxine (30.9%) in the MDD cohort.

#### Treatment’s perceived effectiveness

**Fig. 4:**
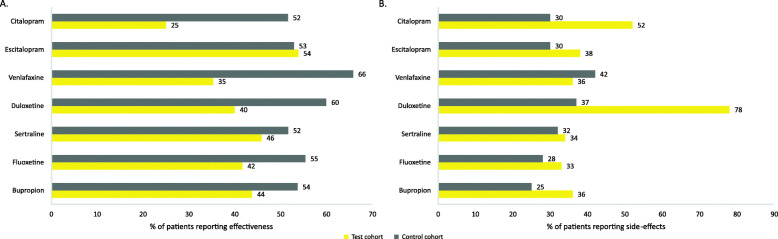
Perceived treatment effectiveness, and treatment side-effects in MDSI and MDD cohorts (**a**) Perceived treatment effectiveness (**b**) Side effects

The proportion of patients who reported “moderate to major perceived effectiveness” with commonly prescribed antidepressant treatments was higher in the MDD cohort compared with the MDSI cohort. The MDSI cohort experienced the lowest perceived effectiveness for citalopram (25.0% vs. 52.0%), venlafaxine (35.0% vs. 66.0%), and duloxetine (40.0% vs. 60.0%) (Fig. 4a).

#### Treatment burden and side-effects

A total of 134 (50.4%) patients in the MDSI cohort and 3070 (25.7%) patients in the MDD cohort reported data for treatment burden and side-effects. Patients in the MDSI cohort more frequently reported side-effects with duloxetine (78.0% vs. 37.0%), citalopram (52.0% vs 30.0%), and bupropion (36.0% vs. 25.0%) compared to those in the MDD cohort. Similar trends were observed for side effects with other treatments (Fig. 4b).

The proportion of patients rating antidepressant treatments as “somewhat to very” burdensome was greater in the MDSI cohort compared to the MDD cohort for sertraline (14.0% vs. 10.0%), venlafaxine (24.0% vs. 14.0%), citalopram (20.0% vs. 12.0%), and duloxetine (22.0% vs. 12.0%); but lesser compared to the MDD cohort for bupropion (8.0% vs. 9.0%), fluoxetine (0% vs. 11.0%), and escitalopram (8.0% vs. 12.0%).

#### Risk factors for suicidal ideation

**Fig. 5:**
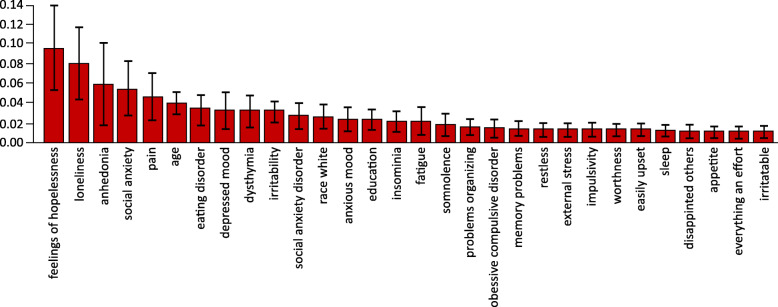
Key risk factors for suicidal ideation in an MDD population

A RF classifier determined the risk factors for SI by predicting whether a patient belonged to the MDSI or MDD cohort. Each variable or feature was assigned an importance value to determine ranking. Variables include demographic characteristics, comorbidities, prompted and unprompted symptoms, and mood map items. Supplementary Figure 2 shows the receiver operating characteristic curve of the RF model. According to the feature importance values obtained from the classifier, feelings of hopelessness (importance value: 0.09 [standard deviation: 0.04]), loneliness (0.08 [0.04]), anhedonia (0.06 [0.04]), social anxiety (0.05 [0.03]) and age (0.04 [0.01]) were the top five predictors of SI in patients diagnosed with MDD in the PLM platform (Fig. 5).

## Discussion

Retrospective analysis of patient-reported data from the PLM depression community showed patients with MDSI are younger, have more comorbidities and higher symptom severity, and experience lower treatment effectiveness but higher treatment side effects.

Among patients with MDD, our results indicated a greater proportion of SI among young adults, with almost 50% of the MDSI cohort being below 35 years of age; as well as among males and patients with a family history of MDD. These findings are consistent with earlier reports and WHO-provided suicide rate data for the US [3, 6, 24]. Han et al. [6] evaluated data of young adults aged 18–25 years from the National Surveys on Drug Use and Health (2009–2015) and reported the mean 2009–2015 annual prevalence of SI among patients with a major depressive episode (MDE) in the US to be 37.3%. Prevalence of SI differs depending on the type of reporting method used. The higher prevalence of SI among 18–25 year old patients in the Han et al. study [6] (37.3%) compared to the MDSI cohort of the present study (28.2%) is probably driven by the difference in reporting method (prompted national survey vs. voluntary web-based platform). In the present study, reporting of SI was anonymous, but patients were not prompted to report any SI-related symptom. Other factors influencing the difference in prevalence of SI could be the larger sample size (*n* = 145,800 vs. *n* = 12,229), age difference (only young adults [18–25 years] vs. all age groups [median age 36 years]), and confirmation of depression (MDE based on DSM-IV vs. self-reported diagnosis of MDD). In line with previous surveys [6, 25–27], the present study reported a greater proportion of women reporting diagnosis of MDD than men regardless of presence of SI.

The greater proportion of young adults in the MDSI cohort was associated with reduced age of onset for depression in this cohort compared to the MDD cohort (< 20 years of age, 72.1% vs. 49.1%). Previous studies have also associated SI with a young age of onset for depression [28–32]. Patients with MDD may be exposed to a greater risk of SI if they do not receive early treatment for MDD [33]. Studies have shown that longer duration of untreated MDD is a critical risk factor for SI in patients. Hence, early screening, diagnosis, and treatment of MDD may contribute to reducing the risk of SI [34]. A novel finding of the current study is that patients in the MDSI cohort reported longer median diagnosis latency than the MDD cohort (4 vs. 2 years), which could be attributed to the lack of mental healthcare-seeking pattern in patients with SI [35, 36] leading to delays in diagnosis and treatment initiation.

In the current study, 68% of the patients reported at least one comorbidity, with the MDSI cohort reporting more comorbidities than the MDD cohort. Additionally, patients with MDSI experienced more comorbid anxiety and substance use disorders than the MDD cohort. These findings were consistent with previous research showing a positive association between number of comorbidities and SI [26, 37, 38]. Furthermore, anxiety and substance use disorder were found to be independent risk factors for suicide and pose a higher risk of suicide attempts [17, 26, 39–41].

Previous research has shown a robust association between depression symptom severity and SI. Two different studies assessed outpatient data employing different methodologies and reported a significant relationship between baseline depression severity and SI [31, 42]. We evaluated symptoms using a three-pronged approach (prompted, MDD-specific, and spontaneous) to enable comprehensive assessment in a standardized manner and obtained similar results. The MDSI cohort exhibited greater prompted and MDD-specific symptom severity (anxious or depressed mood, anhedonia, and irritability), as well as spontaneous patient-reported symptoms pertaining to social isolation, hopelessness, and self-hating thoughts compared to the MDD cohort. These symptoms have been associated with SI previously [31]. However, the symptom severity in the MDSI cohort was greater only at their worst, and not when averaged over time. This unique finding may highlight a specific pattern of symptom evolution in patients with SI. In combination with treatment of depression; patients with SI may require regular tracking of symptom severity; a therapeutic strategy to reduce symptoms at their peak including emotion regulation psychotherapy; and easy access to crisis intervention units when the mitigation strategies fail to control SI. Assessment of depression severity and treatment efficacy in patients with SI needs to focus not only on the average severity but also on the symptom’s maximum severity. Use of digital assessment tools would help patients to report and collect symptoms’ severity data at their peak. However, currently available apps often lack clinical reliability requirements [43].

A greater number of patients in the MDSI cohort experienced severe side effects, lower antidepressant effectiveness, and greater burden from antidepressant use compared to the MDD cohort. The aforementioned outcomes may be interlinked and affect each other. The low perceived treatment effectiveness in the MDSI cohort may be correlated with increased side effect severity; however, such associations were not evaluated. Although patients with MDD and SI require consistent treatment engagement to tackle the complex neuropsychological interplay at hand, their needs to achieve this goal are often unmet due to multiple factors including low adherence to treatment and follow-up [44]. Hence, understanding the patient’s perspective and expectation about treatment is critical to increase patient involvement and continuity of care.

The present study identified young age and feelings of hopelessness, loneliness, anhedonia, and social anxiety to be significantly associated with SI in patients diagnosed with MDD. These risk factors are consistent with previous studies across geographies that employed varying methodologies [27, 45–49]. This result also ties in with the current study’s mood map analysis, which demonstrated increased symptom severity in the MDSI cohort compared to the MDD cohort only during their worse symptom report. Current research continues to look for predictors of suicidal behavior such as biological markers, psychosocial factors and disease characteristics amongst others to improve screening and identification of patients at risk for suicidal behavior. Patient-reported experiences, such as what is available through PLM, allow us to examine associations between various patient characteristics with suicidal behavior from the perspective of the patient. As such, they provide additional data compared to clinician-reported outcomes and add the spontaneously reported patient perspective.

The concept of using online platforms and apps designed for patients with MDD to track their mood has been reported previously [50]. Online communities such as PLM can provide a safe space for individuals who might not receive the support they require from friends, family, and healthcare providers. Moreover, these platforms provide tools to encourage patients to be active and involved in their disease management, thus giving them a sense of control. These benefits have been documented in patients with epilepsy [51, 52]. Sharing symptom and side effect-related information on such platforms may also help in informed decision-making, further benefitting the patient via improved outcomes. Finally, data collected through an online platform provides researchers a different set of information, collected either prospectively or retrospectively, and offers direct access to the patients’ perspective.

The stress-diathesis model acknowledges that suicidal ideation and behavior is the result of a complex pattern of biological and psychological risk factors combined with personal experiences and stressors. The current study showed patients with MDD and SI struggle with more comorbidities and have a different perception of treatment effectiveness and burden. These factors require a tailored approach that may increase adherence to treatment. Critical strategies to improve outcomes in patients with MDD and SI include: screening and detection of high-risk patients; early initiation of pharmacological treatment of MDD in accordance with treatment guidelines [53–56]; psychotherapy specifically designed to enhance patient engagement and manage emotions [15, 16, 57]; and finally, information about on-demand crisis intervention resources to support the patient.

### Limitations

Although standardized methods are routinely utilized for data collection, these methods may not be able to collect spontaneous patient perception data, which is important to bridge the gap between patient perception and evidence. The patient-reported data collected in PLM is of great value to gain access to patient perspectives without the filter of standardized assessment. However, this method is associated with uncertainty regarding their validity and reliability. The cross-sectional nature of this study is also a limitation, as causality cannot be interpreted from the data. Therefore, associations between outcomes such as treatment effectiveness, side effect severity, and burden were not assessed. The requirement for internet access as well as technical competency to engage with the platform may also give rise to bias, as patients with chronic disease often lack these skills [58]. Moreover, participation bias is a concern as research has shown only ~ 1% of members create content, 9% contribute sparingly, and 90% merely observe [59]. However, platforms like PLM do not mandate recording data but rather encourage the patient to take steps gradually. Hence, this limitation is due to the open nature of PLM. Finally, although this study is based on data obtained from a real-world setting, the sample may represent members who differ from the general population.

## Conclusion

The current study highlights that the MDSI population reports worse depressive symptoms, more comorbidities, and lesser perceived effectiveness of antidepressant treatments compared to the MDD population without SI. The current study also demonstrates differences in how patients with MDSI report on their disease experiences which may point to specificities in the psychopathology of MDD in patients with SI compared to MDD patients without SI. Given many of these results are consistent with that obtained from prior, standardized, clinician-rated assessments, this study supports the use of patient-reported data and provides additional information regarding patient perspectives and internal experiences that may be missed with traditional assessments. Further research is required to better understand patient perspectives and identify key leverage points to improve patient satisfaction and clinical outcomes in patients with MDD and SI.

## Supplementary information

**Additional file 1: Supplementary Figure 1.** Mood Map analysis of reported symptoms. (A) Mood map average over time (B) Mood map highest ever severity

**Additional file 2: Supplementary Figure 2.** Receiver operating characteristic curve of the random forest model

**Additional file 3: Supplementary Table 1.** Exclusion criteria

## Data Availability

All the relevant data has been reported in the manuscript. Additional data could be obtained from the authors with an appropriate request.

## Abbreviations

CI: Confidence interval
MDE: Major depressive episode
MDD: Major depressive disorder
MDSI: Major depressive disorder with suicidal ideation
PLM: PatientsLikeMe
SA: Suicide attempt
SI: Suicide ideation
